# Role of Cu in Nanostructural Relationship Between Phase Separation and Deformation-Induced Twinning in Heavily Drawn Non-Equiatomic High-Entropy Alloy Wire

**DOI:** 10.3390/nano15161281

**Published:** 2025-08-20

**Authors:** Sang Hun Shim, Mohsen Saboktakin Rizi, Hossein Minouei, Sun Ig Hong

**Affiliations:** 1 Korea Institute of Materials Science, Changwon 51506, Republic of Korea; shshim@kims.re.kr; 2 Department of Materials Science and Engineering, Chungnam National University, Daejeon 34134, Republic of Korea; mohsen.saboktakin@gmail.com; 3 School of Materials Science and Engineering, Yeungnam University, 280 Daehak-ro, Gyeongsan 38541, Republic of Korea; 4 Graduate Institute of Ferrous Technology (GIFT), Pohang University of Science and Technology, Pohang 37673, Republic of Korea

**Keywords:** phase separation, high-entropy alloy wire, elemental segregation, mechanical property, deformation-induced twining

## Abstract

This study investigates the influence of Cu addition on the nanostructural evolution and mechanical performance of a heavily drawn non-equiatomic CoCu_1.71_FeMnNi high-entropy alloy (HEA) wire. Through systematic microstructural and compositional analysis, we examine how Cu constituent affects phase separation behavior and promotes deformation-induced nano-twinning in another phase counterpart. The designed HEA wire exhibits an elongated ultrafine dual face-centered cubic (fcc) lamella structure (i.e., Co-Fe-rich and Cu-rich phases) that emerges through compositional segregation by spontaneous phase separation from the as-cast state. High-resolution electron microscopy reveals the dislocation wall boundaries stabilized by nanoscale phase interfaces. The cold-drawn CoCu_1.71_FeMnNi wire features an impressive combination of strength and ductility, as well as an ultimate tensile strength of nearly ~2 GPa with an elongation of over ~6%. These findings highlight the critical role of compositional tuning in controlling the ultrafine lamella structure stabilized by spinodal-like phase decomposition, offering a pathway to engineering high-performance HEA wires for advanced structural applications.

## 1. Introduction

Multicomponent high-entropy alloys (HEAs), characterized by their multi-principal element design and simple crystalline phases, have garnered significant attention due to their promising combinations of strength, ductility, and phase stability across a wide range of temperatures [1,2,3]. Among the various HEA systems, the equiatomic CoCrFeMnNi alloy, commonly referred to as the “Cantor alloy”, has been extensively studied as a model face-centered cubic (fcc) system, featuring an outstanding ductility and fracture toughness stemming from the activation of deformation mechanisms such as slip planarity and twinning-induced plasticity (TWIP) at both ambient and cryogenic temperatures [4]. However, the moderate yield strength of the Cantor alloy limits its structural applications, prompting significant efforts to enhance its mechanical performance via compositional tuning, severe plastic deformation, and microstructural engineering [5].

Meanwhile, conventional approaches that involve heavy cold drawing often encounter an intrinsic trade-off between mechanical strength and fracture ductility [6,7]. Wire drawing, as a scalable severe plastic deformation (SPD) technique, introduces high effective strain and microstructural refinement, providing a practical pathway to fabricating ultrahigh-strength metallic fiber or wires [6,7]. In the case of single-phase alloys, the uniformly distributed constituent elements promote both static and dynamic dislocation recovery during the wire drawing processing, which broadens the width of dislocation lamellae and wall boundaries [6,8]. Of course, the spacing of these dislocation wall boundaries is strongly influenced by the stacking fault energy (SFE) of the bulk alloy system, which governs the movement of partial dislocations [9,10]. In low-SFE systems, limited recombination of leading and trailing partials results in dense dislocation structures that enhance strength but typically reduce ductility due to rapid defect accumulation [11].

To overcome these constraints, alternative microstructural strategies, such as introducing phase boundaries through spontaneous phase transformations (e.g., spinodal decomposition or eutectic reactions), have been widely employed in advanced HEA systems [12,13,14,15]. These phase boundaries acted as an efficient barrier for dislocation glide [12], promoting a significant strain partitioning effect between soft and hard domains, thereby enhancing strain hardening and ductility. Eutectic HEAs with Al addition, for instance, have demonstrated enhanced mechanical and functional properties due to their intrinsic microstructural heterogeneity at multiple length scales [16]. On the other hand, the role of composition-driven spinodal decomposition under severe plastic deformation conditions remains poorly understood. In particular, Cu addition to Cantor-variant HEAs is known to alter their thermodynamic stability and SFE together, often promoting microstructural evolution into dual fcc phases via spinodal decomposition and varied dislocation structures during deformation [8,17]. Several studies have reported that Cu-rich variants such as CoCuFeMnNi and CrMnFeNiCu as-cast HEAs exhibit nanoscale Cu-rich and Cu-lean domains that strengthen the alloy through nanoscale interface-derived single dislocation movement and precipitation hardening [5,14,17]. However, phase-separation-derived dual-phase structures not only introduce additional barriers to dislocation motion, but can also initiate different deformation mechanisms within the individual phase domains with varied SFEs. Nonetheless, while Cu is known to increase SFE and suppress the progression of deformation twinning in many single fcc metallic systems, the simultaneous occurrence of accelerated phase separation and deformation twinning under the severe plastic deformation process remains an open question [17].

In this study, we investigated the microstructural interplay between Cu-induced phase separation and deformation-induced twinning structures, as well as related mechanical properties and deformation behavior, in a heavily drawn non-equiatomic CoCu_1.71_FeMnNi high-entropy alloy wire. Through advanced electron microscopy and compositional analysis, we aimed to clarify how the elemental role of Cu addition and severe plastic deformation can collectively influence the evolution of nanostructure and mechanical behavior. The findings are expected to provide new insights into microstructure design strategies for achieving ultrahigh strength–ductility combinations in HEA wire systems.

## 2. Experimental Procedures

### 2.1. Alloy Fabrication and Processing

**Figure 1:**
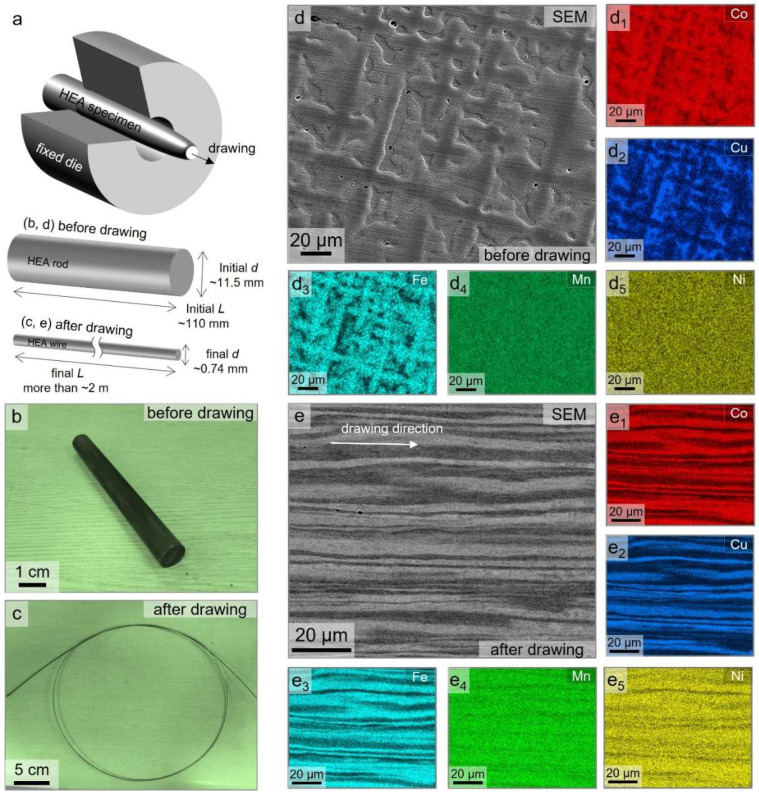
(**a**) The schematic illustration showing the deformation processing and specimen geometries of the initial HEA rod and final wire specimens before/after cold drawing in the present study. (**b**,**c**) The photographs illustrate the specimen geometry (**b**) before and (**c**) after drawing processing. (**d**,**e**) The SEM micrographs and (**d_1_**–**d_5_**,**e_1_**–**e_5_**) corresponding EDS distribution maps illustrating the representative microstructure evolution and elemental separation behaviors of (**d**) homogenized HEA rod and (**e**) as-drawn CoCu_1.71_FeMnNi HEA wire.

An ingot of non-equiatomic high-entropy alloy with a nominal composition of CoCu_1.71_FeMnNi, i.e., (CoFeMnNi)_70_Cu_30_, at.% was prepared by vacuum arc melting under an argon atmosphere. To ensure chemical homogeneity, the ingot was flipped and remelted at least five times. The as-cast alloy was then homogenized at 1200 °C for 24 h in a vacuum furnace, followed by water quenching. The homogenized ingot was machined into cylindrical rods of approximately ~11.5 mm in diameter and ~110 mm in length. Wire drawing was conducted through a series of tungsten carbide dies without intermediate annealing steps, resulting in a final wire diameter of approximately ~0.74 mm and a total drawing length exceeding 2 m. This corresponds to a true strain of ε ≈ 5.5, introducing significant plastic deformation. A schematic of the drawing process and corresponding specimen geometry before and after deformation is illustrated in Figure 1a.

### 2.2. Microstructural Characterization

The microstructures of the homogenized and as-drawn samples were examined using scanning electron microscopy (SEM, ZEISS-Merlin, Ettlingen, Germany) equipped with energy-dispersive X-ray spectroscopy (EDS, Energy_X-MaxN, Oxford, UK) and an electron backscatter diffraction (EBSD, Oxford-NordlysNano, High Wycombe, UK) detector. Phase identification and orientation mapping were conducted using EBSD, with step sizes of 50 nm. For higher-resolution analysis, transmission electron microscopy (TEM) and scanning transmission electron microscopy (STEM) were performed using a JEOL ARM-200F microscope, Tokyo, Japan, operated at 200 kV. Elemental distributions were obtained by STEM-EDS mapping, Ettlingen, Germany. Thin foils for TEM were prepared by focused ion beam (FIB, SEIKI-SMI3050SE, Chiba, Japan) milling from longitudinal sections parallel to the drawing direction from the central region of the wire specimens.

### 2.3. Mechanical Testing

Room-temperature uniaxial tensile tests were carried out on as-drawn wires using a universal materials testing machine (US/SSTM, UNITED, Warren, MI, USA) at an initial strain rate of 10^−3^ s^−1^. Tensile specimens were cut from wire segments and prepared with a gauge length of 25 mm. The engineering stress–strain response was recorded, and fracture surfaces were analyzed via SEM to determine the failure mechanism.

## 3. Results and Discussion

### 3.1. Microstructural Analysis

Figure 1 illustrates the overall geometry and microstructural evolution of the rod-type CoCu_1.71_FeMnNi ingot and fiber-like drawn wire before/after the wire drawing processing. As shown in Figure 1a, the starting material, with an initial diameter of 11.5 mm, was processed into a 0.74 mm diameter wire, corresponding to a true strain of approximately ε ≈ 5.5. Photographs of the HEA rods and cold-drawn wire are shown in Figure 1b,c. The cold drawing was carried out continuously without intermediate annealing, allowing the alloy wire to accumulate significant plastic strain and enabling microstructural refinement and phase separation. Figure 1d shows the SEM image of the homogenized alloy (i.e., HEA rods), indicating pattern-like segregated features, indicative of compositional heterogeneity at the microscale. The corresponding EDS elemental maps, as shown in Figure 1(d_1_–d_5_), reveal the distinct dual-phase structure, comprising (1) Cu-rich and (2) Co-Fe-rich fcc phases stabilized by spontaneous phase separation forming chemically segregated domains even after homogenization. This is consistent with prior findings that such compositional partitioning can persist in Cu-containing HEAs due to thermodynamic instability and the positive mixing enthalpy (ΔH_mix_) between Cu and other transition metals, such as Fe and Co [5,14]. After the cold-drawing process shown in Figure 1e, the microstructure became highly elongated and aligned parallel to the drawing direction. This directional morphology indicates substantial plastic flow, and the layered contrast reflects the mechanical imprint of phase-separated regions. The EDS mapping results (Figure 1(e_1_–e_5_)) confirm the persistence of Cu-rich and Cu-depleted (i.e., Fe-Co-rich) domains, and detailed chemical compositions of the Cu-rich and Co-Fe-rich dual phases from the rod and wire specimens are listed in Table 1. (Hereafter, they are referred to as “Co-Fe-rich fcc1” and “Cu-rich fcc2”, respectively.) This indicates the pronounced average chemical composition for Co-Fe-rich fcc1 and Cu-rich fcc2 and phases, regardless of the drawing process. The average chemical composition of both dual-phase structures was approximately Co22-24Cu9-10Fe26−27Mn19−22Ni20 and Co6−7Cu48−50Fe8−10Mn20−22Ni12−15 for Co-Fe-rich fcc1 and Cu-rich fcc2 phase constituents before/after the cold drawing process. This suggests that there exists no noticeable compositional deviation caused by heavy deformation processing without heat treatment. The segregation behavior between Cu and the Co and Fe elemental groups was still found to be predominant, suggesting that the internal driving force for mixing elemental constituents together was higher than that for occurring the phase separation, albeit with the heavy deformation process (i.e., cold drawing).

**Table 1:** Average chemical composition of dual fcc structure in CoCu_1.71_FeMnNi HEA rod and as-drawn wires (i.e., before/after cold drawing process).

Specimen	Phase	Co (at %)	Cu (at %)	Fe (at %)	Mn (at %)	Ni (at %)
HEA rod	Co-Fe-rich fcc1	~22.5 ± 2.5	~8.9 ± 1.7	~27.7 ± 1.8	~21.6 ± 1.7	~19.3 ± 2.4
	Cu-rich fcc2	~6.3 ± 0.6	~50.0 ± 3.1	~8.4 ± 1.5	~22.4 ± 0.9	~12.9 ± 1.7
HEA wire	Co-Fe-rich fcc1	~24.1 ± 1.1	~10.7 ± 2.8	~26.4 ± 2.1	~18.7 ± 2.1	~20.1 ± 1.4
	Cu-rich fcc2	~7.4 ± 1.0	~47.5 ± 2.4	~10.3 ± 2.7	~19.6 ± 1.8	~15.2 ± 1.1

The presence of a dual fcc phase structure in the CoCu_1.71_FeMnNi specimen, consisting of Cu-rich and Co-Fe-rich regions, is likely attributed to the spinodal-like phase decomposition occurring because of uphill diffusion, caused by a relatively high contribution of mixing enthalpy rather than mixing entropy terms. According to Shim et al. [5], this modulated phase separation occurs due to reductions in lattice strain and interface energies, facilitated by selective atomic redistribution into Cu-rich and Co-Fe-rich phase domains. The close lattice constants of the two phases allow for the formation of coherent interfaces, which appear as continuous fcc diffraction patterns in XRD analysis from elsewhere [5,5]. The development of such a nanoscale modulation is energetically favorable in multicomponent alloys, particularly when Cu is enriched beyond equiatomic atomic concentrations. This microstructural pattern is distinct from dendritic segregation and represents a stable, thermodynamically driven phenomenon that persists through high-strain processing.

**Figure 2:**
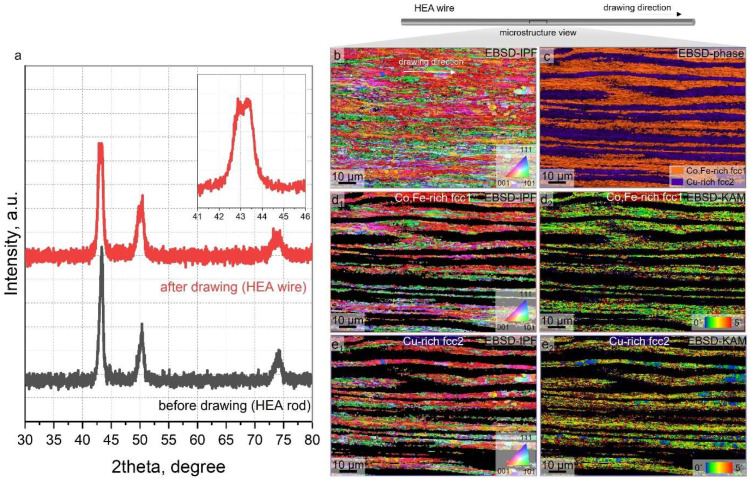
(**a**) Typical XRD patterns taken from homogenized HEA ingot and as-drawn HEA wire. (**b**) EBSD inverse-pole figure (IPF), and (**c**) phase micrographs showing the deformed microstructure of as-drawn HEA wire. (**d_1_**,**e_1_**) Corresponding EBSD-IPF and (**d_2_**,**e_2_**) EBSD-KAM micrographs elucidating the microstructure evolution and dislocation accumulation within the (**d**) Co,Fe-rich fcc1 and (**e**) Cu-rich fcc2 phases.

**Figure 3:**
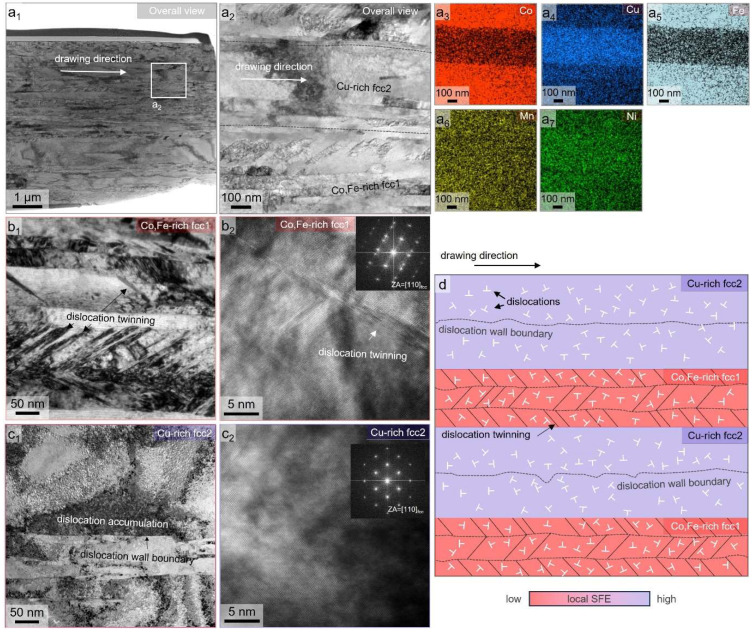
(**a_1_**,**a_2_**) Bright-field STEM micrographs and (**a_3_**–**a_7_**) corresponding EDS distribution maps indicating the representative nanostructure and elemental segregation behavior of as-drawn CoCu_1.71_FeMnNi HEA wire. (**b_1_**,**b_2_**,**c_1_**,**c_2_**) The enlarged bright-field and high-resolution TEM micrographs within the (**b**) Co,Fe-rich fcc1 and (**c**) Cu-rich fcc2 phases, highlighting the presence of deformation-induced twinning structure or dislocation structure stemming from heavy deformation of cold-drawn HEA wire. (**d**) The schematic diagram illustrating the micro/nanostructure evolution observed by EBSD and TEM analyses.

Figure 2a shows the typical XRD diffraction patterns obtained from both the homogenized HEA ingot and the as-drawn wire. In both conditions, the diffraction peaks primarily display an overlapped fcc pattern, originating from the dual-phase structure of Co-Fe-rich fcc1 and Cu-rich fcc2. Although these phases are compositionally distinct, they exhibit very similar lattice constants due to the presence of common elements such as Mn and Ni, which are uniformly distributed across both fcc domains [5]. This compositional convergence results in lattice parameters that are too close to resolve as fully distinct peaks, hence appearing as slightly overlapped or single set-like peaks in the XRD pattern. However, careful observation reveals subtle peak splitting, particularly in the as-drawn condition, which supports the presence of two fcc phases with closely related but non-identical lattice parameters. These findings are further confirmed by the lattice constant measurements derived from the FFT analysis in Figure 3, which show minor but consistent differences between fcc1 and fcc2. This reinforces their structural duality despite the apparent peak merging in XRD. Additionally, the microstructure presented in Figure 1 shows a characteristic compositional modulation in the as-drawn wire, consistent with spinodal decomposition. The alignment between this modulated microstructure and the subtle peak splitting observed in XRD provides strong, complementary evidence for spinodal-type phase separation in the present HEA system. Nevertheless, the XRD peaks become more broadened after the cold drawing process, suggesting that heavy deformation processing can induce the formation of a large dislocation density and nanoscale subgrain boundary within the crystal structure of each dual-phase structure, which leads to peak broadening [5,18]. In addition, the slightly split XRD peaks also support that the formation of a dual-phase structure is energetically stable.

To further investigate the structural evolution induced by cold drawing deformation, EBSD phase and orientation mapping were performed on the drawn CoCu_1.71_FeMnNi wire. Figure 2a shows the EBSD inverse pole figure (IPF) map along the wire axis, indicating elongated grains and subgrain structures aligned with the drawing direction. This strong texture, characteristic of uniaxial deformation, indicates significant strain accumulation and confirms the presence of a lamellar structure aligned with the drawing direction—consistent with previous reports on spinodal decomposition in Cu-containing HEAs [19]. The phase identification map confirms the presence of two fcc phases, i.e., Co-Fe-rich fcc1 and Cu-rich fcc2, respectively, consistent with the compositional contrast shown in Figure 1. This dual-phase structure, sustained even after heavy deformation, reflects a chemically driven phase separation stabilized by differences in thermodynamic interactions and elemental affinities. Kernel Average Misorientation (KAM) maps (Figure 2c,d) reveal that the Co-Fe-rich fcc1 phase exhibits a higher average KAM value (~2.75 ± 1.47°) than the Cu-rich fcc2 phase (~2.39 ± 1.59°), suggesting a greater strain localization and dislocation density in the former. This discrepancy is closely linked to variations in stacking fault energy (SFE), which is strongly composition-dependent. Prior studies have shown that elements like Co and Fe tend to reduce SFE [19], promoting dislocation dissociation and deformation twinning, while Cu increases SFE and tends to stabilize perfect dislocation glide. Wei et al. [20] demonstrated that in metastable HEA systems, Co-rich regions with a low SFE facilitate stacking fault embryo formation and twinning or even martensitic transformation under strain. Conversely, Cu addition delays such transformations by raising the SFE and promoting fcc phase stability. In our system, the measured compositions of the dual-phase domains—approximately Co_24_Cu_9_Fe_26_Mn_20_Ni_20_ for fcc1 and Co_7_Cu_50_Fe_10_Mn_22_Ni_15_ for fcc2 (at%)—suggest a clear contrast in elemental content and, accordingly, in SFE behavior. Although a precise numerical estimate of SFE difference is not provided, this compositional divergence strongly implies that fcc1 has a lower SFE than fcc2, which is supported by differences in their dislocation activity and average KAM values.

The observed microstructure supports the hypothesis that compositional partitioning induced by Cu enrichment leads to elemental dissipation and creates a microscale heterostructure with a varying local SFE, enabling phase-specific deformation responses. The fcc1 domain is expected to be twinning-prone due to its lower SFE, while the fcc2 phase domain with a higher Cu content and SFE primarily accommodates plasticity through dislocation glide, planar slip, and potential stacking fault formation. This dual-phase interaction enables the simultaneous activation of twinning and dislocation-mediated deformation. The interplay between these mechanisms allows for efficient strain accommodation through interface-mediated hardening, contributing to the observed strength–ductility synergy in the heavily drawn HEA wire.

Next, to further examine the nanoscale structural evolution in the cold-drawn CoCu_1.71_FeMnNi HEA wire, STEM-EDS elemental mapping and TEM imaging were performed on thin foils prepared out-of-plane to the drawing direction. Figure 3(a_1_–a_7_) displays the high-angle annular bright-field (HAABF) STEM image with (Figure 3(a_1_,a_2_)) different magnification views and corresponding EDS maps for (Figure 3(a_3_)) Co, (Figure 3(a_4_)) Cu, (Figure 3(a_5_)) Fe, (Figure 3(a_6_)) Mn, and (Figure 3(a_7_)) Ni, respectively. From the results, elemental segregation observed at the nanometer scale (Figure 3) is consistent with spinodal-like phase separation into Cu-rich and Co-Fe-rich regions. The spatial partitioning of Cu is particularly prominent in the central region in Figure 3(a_2_), while the constituents of Co and Fe are reversed, forming a chemically heterogeneous dual-phase fcc structure. In addition, it is clearly confirmed that the phase interface of the dual-phase structure coincides with the dislocation wall boundaries. The designated phase interface from the EDS mapping results (Figure 3(a_3_–a_7_)) is marked by a dashed black line, as shown in Figure 3(a_2_). It should be noted that the coincident boundary of the phase interface and dislocation wall boundaries in Figure 3(a_2_) slightly differs. This is because the phase interface of the dual-phase structure has boundary curvature into the in-plane direction of FIB specimens, which blurs the specific location of the phase interface [21]. In Figure 3(a_1_), the localized regions of the STEM image exhibit different contrasts and substructural characters. This regional variation is not due to sample thickness, but rather reflects the microstructural differences between the Co-Fe-rich fcc1 and Cu-rich fcc2 phase domains. The finer and coarser subgrain structures align with these phase domains and suggest that differences in SFE influence the development and morphology of dislocation wall boundaries. Specifically, lower-SFE regions (fcc1) tend to form highly dense twin-containing structures, while higher-SFE regions (fcc2) favor more planar and dense dislocation walls. To highlight this phase-specific difference, an enlarged view is added in Figure 3(a_2_), showing clearly that the Co-Fe-rich region accommodates inclined twin structures, while the Cu-rich region shows refined dislocation wall structures. This observation reinforces the phase-dependent deformation behavior discussed earlier and is now explicitly addressed in the revised text for clarity.

In view of this, the upper and lower regions of the Co-Fe-rich fcc1 phase in Figure 3(a_2_) reveal the presence of an inclined deformation-induced twinning structure, whereas none of such structure is found in the Cu-rich fcc2 phase region. Accordingly, there exists a different trend in nanostructure formation with respect to their chemical compositions. The bright-field TEM micrographs (Figure 3b,c) reveal a highly deformed substructure composed of elongated grains and dense dislocation walls. Within this deformation substructure, numerous deformation-induced twining structures are observed within the Co-Fe-rich fcc1 phase (Figure 3b). These features are predominantly located within the Co-Fe-rich fcc1 phase domain, supporting the interpretation that the reduced SFE in these regions facilitates the twin nucleation. In contrast, the Cu-rich fcc2 region shows no such twining structures, but there exists a well-developed sub-boundary with dislocation accumulation (Figure 3c), suggesting that dislocation glide is the dominant mechanism in this phase region with a relatively higher SFE. In addition, the calculated lattice parameters obtained from the fast Fourier transform (FFT) patterns in the Co-Fe-rich fcc1 and Cu-rich fcc2 phases are estimated to be ~0.3578 and ~0.3554 nm, respectively. The close lattice parameters are likely to originate from a spinodal-like dual-phase lamella structure with different chemical behavior, whose structure can be sustained after a heavy cold drawing process. Indeed, the differences in lattice parameters are quite indistinguishable, and this trend is consistent with the split XRD peak result in Figure 2a.

Notably, twin boundaries and dislocation walls are often found adjacent to phase interfaces, indicating a strong coupling between phase boundaries and deformation modes. These interfaces not only act as effective barriers to dislocation motion but also accommodate strain mismatch between adjacent phase domains, promoting plastic compatibility during deformation. This phenomenon aligns with observations in other dual-phase and spinodal-decomposed fcc systems, where the strain partitioning and heterogeneous microstructure enhance work hardening capacity [14]. Overall, the TEM and STEM analyses in Figure 3a–c provide direct evidence of nanoscale compositional heterogeneity and phase-specific deformation behavior. This complex microstructure evolution is schematically illustrated in Figure 3d. The coexistence of dislocation accumulation and twin-prone regions supports a multi-mode deformation mechanism, in which phase boundaries, dislocation walls, and twins jointly contribute to the strength–ductility synergy of the HEA wire. This dual mechanism is particularly important in heavily drawn wires, where uniform deformation and the suppression of early failure are essential for mechanical reliability.

### 3.2. Mechanical Properties

**Figure 4:**
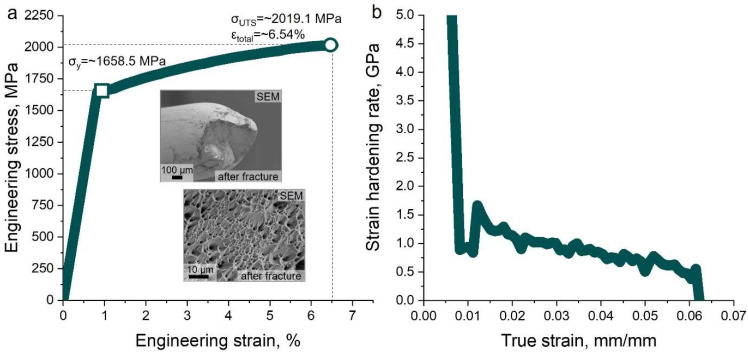
(**a**) Uniaxial tensile engineering stress–strain curve, and (**b**) converted strain hardening rate versus true strain curve of present as-drawn CoCu_1.71_FeMnNi HEA wire. Insets in (**a**) exhibits the SEM fractography micrographs enclosing the presence of necking and dimple structure, supporting the ductile fracture for as-drawn Cu30 HEA wire.

Figure 4a shows the engineering stress–strain curve of the heavily drawn CoCu_1.71_FeMnNi HEA wire. The present HEA wire exhibits a high ultimate tensile strength (UTS) of approximately ~2019.1 ± 62.8 MPa and a corresponding yield strength of ~1658.5 ± 46.1 MPa, with a total elongation of about ~6.54%. This combination of strength and moderate ductility places the material alongside other fcc-based HEAs processed via severe plastic deformation, surpassing many previously reported drawn wires in both equiatomic and non-equiatomic systems [5,8,14].

The extraordinarily high strength is attributed to several concurrent mechanisms activated during the cold drawing process. This heavy deformation processing involves grain elongation and/or refinement, which further increases the surface area of the grain boundary and contributes to enhancing Hall–Petch-type strengthening. Additionally, the formation of dislocation walls obstructs further dislocation glide, thereby enhancing resistance to plastic deformation. The phase interface between Co-Fe-rich and Cu-rich fcc phase domains also plays a crucial role, acting not only as an effective barrier to dislocation motion, but also suppressing growth in the dislocation wall boundary of the dual-phase domains interacting with each other. Then, it is expected that the narrow-spaced dual-phase lamella structure can simultaneously affect the strengthening behavior and strain hardening rate as well.

Figure 4b shows the corresponding strain hardening rate (*dσ*/*dε*) versus the true strain curve. A high initial hardening rate over ~5 GPa is observed, which gradually declines with an increasing true strain, but remains positive until failure, indicating continuous strain hardening throughout the tensile process. This behavior is consistent with microstructural observations of evolving dislocation structures and twin formation during cold drawing, especially in the Co-Fe-rich fcc1 phase (Figure 3). The insets in Figure 4a reveal the SEM micrograph, highlighting the fractography of the present HEA wire after tensile fracture, indicating the pronounced dimple structure. This suggests that the present HEA wire fabricated by the heavy cold drawing process undergoes the ductile fracture mode prior to failure. Despite its high strength, the preservation of a ductile fracture morphology highlights the inherent ability to maintain mechanical integrity under the tensile loading of HEA wires, a direct consequence of the multi-mode deformation mechanism described earlier. Together, these results confirm that the combination of Cu-induced phase separation and deformation-induced twinning, facilitated by high-strain wire drawing, produces a refined dual-phase microstructure capable of balancing strength and ductility. This unique alloy composition and simple processing strategy show that the present CoCu_1.71_FeMnNi HEA wire is a promising candidate for advanced structural applications requiring an ultrahigh strength without sacrificing elongation.

### 3.3. Deformation Mechanism

**Figure 5:**
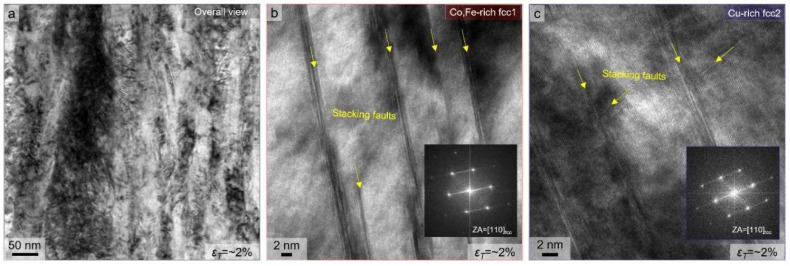
Deformed microstructure of as-drawn HEA wire after interrupt strain (i.e., *ε_T_* = ~2%) from (**a**) overall view and (**b**,**c**) enlarged views within (**b**) Co,Fe-rich fcc1 and (**c**) Cu-rich fcc2 phases, respectively.

To gain insight into the deformation mechanisms occurring after tensile loading, thin foils were extracted by FIB milling from the interrupted tensile specimens strained to approximately ~2%. The bright-field TEM images from these samples are presented in Figure 5a–c. Even at this low strain level, a high density of stacking faults, incipient deformation twins, and dislocation entanglement is observed in overall views (Figure 5a), indicating that significant plastic activity is already underway during the initial elastic–plastic transition. In particular, the stacking faults appear as planar defects across both the fcc1 and fcc2 regions (Figure 5b,c), suggesting that partial dislocation motion is readily activated upon yielding. This is consistent with the relatively low average SFE of the alloy system, especially in the Co-Fe-rich (i.e., Cu-depleted) fcc1 domain [17]. Short and narrow stacking fault bundles are also visible within fcc2 grains (Figure 5c), confirming that both phase domains are dynamically strengthened by the progression of stacking faults after tensile deformation. The formation of deformation-induced twins caused by cold drawing enhances the strain hardening rate at early plastic strain by subdividing grains and blocking dislocation glide. Meanwhile, the presence of stacking faults is also observed in fcc2, which acts as a slip barrier. Generally, the coincidence of soft and hard phase constituents can be strengthened by load transfer, where the robust phase provides mechanical hardening into the soft-phase domain to simultaneously accommodate plastic deformation [22]. From this viewpoint, the early activation of stacking faults in dual-phase structures with different SFEs supports a multi-mode deformation pathway, where the plastic compatibility between phase-separated domains is maintained from the onset of deformation.

These observations reinforce the conclusion that the Cu-induced phase-separated microstructure facilitates the simultaneous operation of dislocation glide, stacking fault formation, and twinning, contributing to the alloy’s sustained work hardening behavior and high mechanical performance (as indicated in Figure 4b). The initiation of deformation mechanisms at low strain further underscores the role of local SFE variations and nanoscale structural heterogeneity in enabling efficient strain accommodation.

## 4. Conclusions

In this study, the effects of Cu addition on the nanostructural evolution and deformation mechanisms of a heavily drawn non-equiatomic CoCu_1.71_FeMnNi high-entropy alloy (HEA) wire are systematically investigated. The results demonstrate that compositional tuning through Cu enrichment effectively modulates phase stability and deformation mechanisms, enabling the design of non-equiatomic HEAs with superior strength–ductility combinations. The findings offer valuable insight into the development of high-performance, wire-processed HEA systems for structural and functional applications. The key findings are summarized as follows:

1. Severe plastic deformation via cold drawing introduced a high true strain (~5.5), leading to significant microstructural refinement and spinodal-like phase separation into dual fcc phases, that is, Co-Fe-rich fcc1 and Cu-rich fcc2 phases—without heat treatment.
2. Experimentally, different degrees of dislocation accumulation and distinct deformation structure appearances resulted in the dual-phase structure, which was caused by Cu-induced phase-separation-driven stacking fault energy (SFE) variations.
3. The Co-Fe-rich fcc1 (Cu-depleted) phase, having a lower SFE, accommodated a higher dislocation density with a deformation twinning and stacking faults structure, while the Cu-rich fcc2 phase deformed mainly via dislocation glide. This complementary deformation response promoted strain partitioning and plastic compatibility across phase boundaries.
4. The cold-drawn HEA wire exhibited an exceptional ultimate tensile strength of nearly ~2 GPa with a total elongation of ~6.5%, attributed to a synergistic combination of twinning-induced plasticity, dislocation hardening, and interface strengthening.
5. Interrupted tensile tests revealed that the presence of stacking faults was activated at very early stages of deformation (interrupting strain of ~2%), confirming the onset of multi-mode plasticity from the beginning of loading.

## Data Availability

Data available on request due to restrictions.
